# Augur: a bioinformatics toolkit for phylogenetic analyses of human pathogens

**DOI:** 10.21105/joss.02906

**Published:** 2021-01-07

**Authors:** John Huddleston, James Hadfield, Thomas R. Sibley, Jover Lee, Kairsten Fay, Misja Ilcisin, Elias Harkins, Trevor Bedford, Richard A. Neher, Emma B. Hodcroft

**Affiliations:** 1 Molecular and Cellular Biology Program, University of Washington, Seattle, WA, USA; 2 Vaccine and Infectious Disease Division, Fred Hutchinson Cancer Research Center, Seattle, WA, USA; 3 Biozentrum, University of Basel, Basel, Switzerland; 4 Swiss Institute of Bioinformatics, Basel, Switzerland; 5 Institute of Social and Preventive Medicine, University of Bern, Bern, Switzerland

## Abstract

The analysis of human pathogens requires a diverse collection of bioinformatics tools. These tools include standard genomic and phylogenetic software and custom software developed to handle the relatively numerous and short genomes of viruses and bacteria. Researchers increasingly depend on the outputs of these tools to infer transmission dynamics of human diseases and make actionable recommendations to public health officials (Black et al., 2020; Gardy et al., 2015). In order to enable real-time analyses of pathogen evolution, bioinformatics tools must scale rapidly with the number of samples and be flexible enough to adapt to a variety of questions and organisms. To meet these needs, we developed Augur, a bioinformatics toolkit designed for phylogenetic analyses of human pathogens.

Augur originally existed as an internal component of the nextflu (Neher & Bedford, 2015) and Nextstrain (Hadfield et al., 2018) applications. As a component of nextflu, Augur consisted of a single monolithic Python script that performed most operations in memory. This script prepared a subset of seasonal influenza sequences and metadata and then processed those data to produce an annotated phylogeny for visualization in the nextflu web application. When Nextstrain replaced nextflu and expanded to support multiple viral and bacterial pathogens, each pathogen received its own copy of the original script. The resulting redundancy of these large scripts complicated efforts to debug analyses, add new features for all pathogens, and add support for new pathogens. Critically, this software architecture led to long-lived, divergent branches of untested code in version control that Nextstrain team members could not confidently merge without potentially breaking existing analyses.

## Implementation

To address these issues, we refactored the original Augur scripts into a toolkit of individual subcommands wrapped by a single command line executable, augur. With this approach, we followed the pattern established by samtools (Li et al., 2009) and bcftools (Li, 2011) where subcommands perform single, tightly-scoped tasks (e.g., “view,” “sort,” “merge,” etc.) that can be chained together in bioinformatics pipelines. We migrated or rewrote the existing functionality of the original Augur scripts into appropriate corresponding Augur subcommands. To enable interoperability with existing bioinformatics tools, we designed subcommands to accept inputs and produce outputs in standard bioinformatics file formats wherever possible. For example, we represented all raw sequence data in FASTA format, alignments in either FASTA or VCF format, and phylogenies in Newick format. To handle the common case where a standard file format could not represent some or all of the outputs produced by an Augur command, we implemented a lightweight JSON schema to store the remaining data. The “node data” JSON format represents one such Augur-specific file format that supports arbitrary annotations of phylogenies indexed by the name assigned to internal nodes or tips. To provide a standard interface for our own analyses, we also designed several Augur subcommands to wrap existing bioinformatics tools including augur align (mafft (Katoh et al., 2002)) and augur tree (FastTree (Price, 2010), RAxML (Stamatakis, 2014), and IQ-TREE (Nguyen et al., 2014)). Many commands including augur refine, traits and ancestral make extensive use of TreeTime (Sagulenko et al., 2018) to provide time-scaled phylogenetic trees or further annotate the phylogeny.

**Figure 1:**
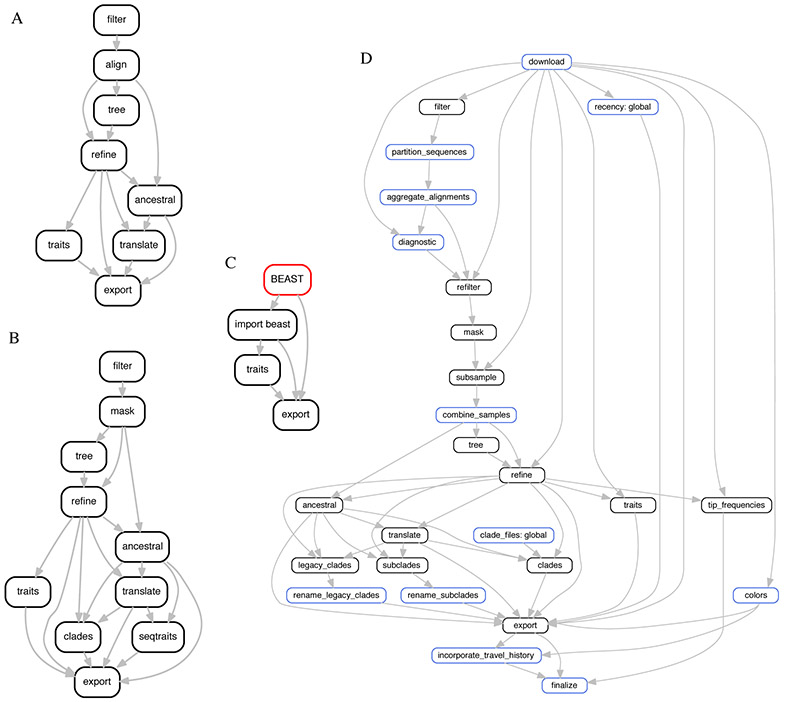
Example workflows composed with Snakemake from Augur commands for A) Zika virus, B) tuberculosis, C) a BEAST analysis, and D) the Nextstrain SARS-CoV-2 pipeline as of 2020-11-27. Each node in the workflow graph represents a command that performs a specific part of the analysis (e.g., aligning sequences, building a tree, etc.) with Augur commands in black, external software in red, and custom scripts in blue. A typical workflow starts by filtering sequences and metadata to a desired subset for analysis followed by inference of a phylogeny, annotation of that phylogeny, and export of the annotated phylogeny to a JSON that can be viewed on Nextstrain. Workflows for viral (A) and bacterial (B) pathogens follow a similar structure but also support custom pathogen-specific steps. Augur’s modularity enables workflows that build on outputs from other tools in the field like BEAST (C) as well as more complicated analyses such as that behind Nextstrain’s daily SARS-CoV-2 builds (D) which often require custom scripts to perform analysis-specific steps. Multiple outgoing edges from a single node represent opportunities to run the workflow in parallel. See the full workflows behind A, B, and D at https://github.com/nextstrain/zika-tutorial, https://github.com/nextstrain/tb, and https://github.com/nextstrain/ncov.

By implementing the core components of Augur as a command line tool, we were able to rewrite our existing pathogen analyses as straightforward bioinformatics workflows using existing workflow management software like Snakemake (Köster & Rahmann, 2012). Most pathogen workflows begin with user-curated sequences in a FASTA file (e.g., sequences.fas ta) and metadata describing each sequence in a tab-delimited text file (e.g., metadata. tsv). Users can apply a series of Augur commands and other standard bioinformatics tools to these files to create annotated phylogenies that can be viewed in Auspice, the web application that serves Nextstrain (Figure 1). This approach allows users to leverage the distributed computing abilities of workflow managers to run multiple steps of the workflow in parallel and also run individual commands that support multiprocessing in parallel. Further, the Augur modules can be easily recombined both with each other and with user-generated scripts to flexibly address the differing questions and restrictions posed by a variety of human pathogens.

The modular Augur interface has enabled phylogenetic and genomic epidemiological analyses by academic researchers, public health laboratories, and private companies. Most recently, these tools have supported the real-time tracking of SARS-CoV-2 evolution at global and local scales (Alm et al., 2020; Bedford et al., 2020; The Nextstrain Team, 2020). This success has attracted contributions from the open source community that have allowed us to improve Augur’s functionality, documentation, and test coverage. To facilitate Augur’s continued use as part of wider bioinformatics pipelines in public health, we have committed to work with and contribute to open data standards such as PHA4GE (Griffiths et al., 2020) and follow recommendations for open pathogen genomic analyses (Black et al., 2020). Augur can be installed from PyPI (nextstrain-augur) and Bioconda (augur). See the full documentation for more details about how to use or contribute to development of Augur.
